# A Clinical Case of Aneurysmal Dilatation of the Aortic Arch Distal to the Origin of an Aberrant Right Subclavian Artery Treated with Castor Single-Branch Stent Graft Implantation and Right Carotid-Subclavian Bypass

**DOI:** 10.3390/jcdd12070251

**Published:** 2025-06-29

**Authors:** Antonio Rizza, Silvia Di Sibio, Angela Buonpane, Giancarlo Trimarchi, Marta Casula, Michele Murzi, Pierandrea Farneti, Cataldo Palmieri, Marco Solinas, Sergio Berti

**Affiliations:** 1 Ospedale del Cuore, Fondazione Toscana “G. Monasterio”, 54100 Massa, Italy; 2 Department of Cardiovascular Sciences, Fondazione Policlinico Universitario A. Gemelli IRCCS, Università Cattolica Sacro Cuore, 00168 Rome, Italy; 3 Interdisciplinary Center for Health Sciences, Scuola Superiore Sant’Anna, 56127 Pisa, Italy; 4 Clinical Experimental Cardiology, Clinical and Interventional Cardiology, University of Sassari, 07100 Sassari, Italy

**Keywords:** aberrant right subclavian artery (ARSA), Castor single-branch stent graft (CSBSG), hybrid procedure, Aortic Team

## Abstract

Advancements in endovascular stent graft design have enabled the treatment of distal aortic arch pathologies. However, the length of the proximal landing zone remains a limitation, especially with vascular anomalies like an aberrant right subclavian artery (ARSA) posing additional challenges. A 78-year-old patient underwent computed tomography angiography (CTA), which revealed progressive enlargement of a distal aortic arch aneurysm located beyond an ARSA that coursed between the esophagus and trachea. Following evaluation by the multidisciplinary Aortic Team, a hybrid procedure was planned. A right carotid-to-ARSA bypass was performed and a Castor single-branched stent graft (CSBSG) was deployed in the aortic arch with its side branch directed into the left subclavian artery (LSA), thereby covering the origin of the ARSA. To prevent a type II endoleak, plug embolization of the ARSA origin was subsequently performed. CSBSG is a feasible treatment for distal aortic arch aneurysms, even in the presence of vascular anomalies such as ARSA.

## 1. Introduction

Thoracic endovascular aortic repair (TEVAR) has significantly improved the management of complex aortic arch pathologies, offering a less invasive alternative to open surgery. However, the presence of anatomical anomalies such as an aberrant right subclavian artery (ARSA) can complicate procedural planning and execution. ARSA, a congenital vascular variant in which the right subclavian artery (RSA) arises distal to the left subclavian artery (LSA), occurs in approximately 0.16% to 1.8–2% of the population [1,2]. While often asymptomatic, its atypical origin and retroesophageal course may limit available proximal landing zones for endograft fixation and increase the risk of neurological or ischemic complications if not adequately addressed.

In recent years, the development of branched stent graft technology, including the Castor single-branched stent graft (CSBSG), has enabled targeted revascularization of critical arch vessels during endovascular procedures. This prosthesis, designed with an integrated side branch, allows for the preservation of perfusion to the LSA, even when its origin must be covered to achieve adequate sealing of the aortic arch.

We present a case in which a hybrid approach was employed to treat a distal aortic arch aneurysm in a patient with ARSA.

## 2. Case Presentation

A 78-year-old man, who had previously undergone ascending aorta replacement and aortic valve shaving in 2022, was found during follow-up computed tomography angiography (CTA) to have enlargement of a distal aortic arch aneurysm (58 × 57 mm). Additionally, CTA revealed the presence of an ARSA originating distal to the LSA, coursing posteriorly between the esophagus and trachea.

The patient’s medical history was notable for hypertension, dyslipidemia, and a family history of coronary artery disease and cerebrovascular disorders.

**Figure 1:**
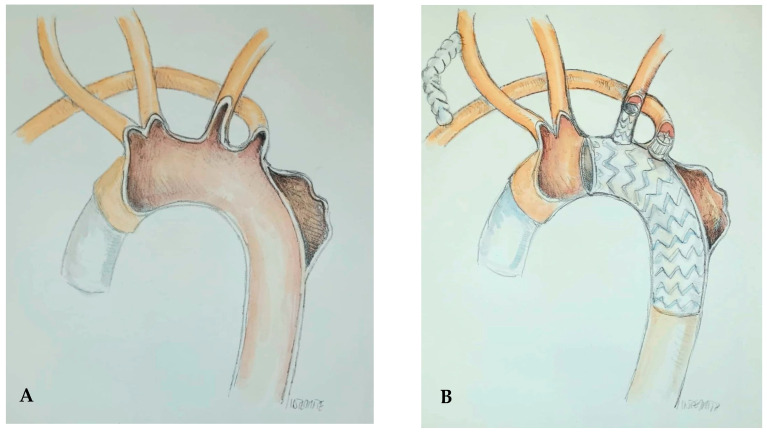
Graphical representation of the anatomy of the aberrant right subclavian artery (ARSA) and aneurysm: pre-procedure (**A**) and post-prosthesis implantation with a Castor device, including carotid-subclavian bypass and plug implantation at the origin of ARSA (**B**).

Following multidisciplinary discussion within the Aortic Team, a personalized hybrid strategy was planned to manage the aortic arch aneurysm in the context of ARSA. A schematic representation of the procedure is shown in Figure 1.

Initially, the right common carotid artery (RCCA) and the ARSA were exposed through a cervical incision. An 8 mm Dacron graft was anastomosed end-to-side to the RCCA and then to the ARSA to create a right carotid-subclavian bypass. The right and left common femoral arteries (RCFA and LCFA) were accessed with 6 Fr sheaths. The right and left brachial arteries (RBA and LBA) were cannulated using 8 Fr and 10 Fr sheaths, respectively.

A pre-closure technique was performed in the RCFA using two Perclose™ ProStyle™ suture-mediated closure systems (Abbott Vascular, Santa Clara, CA, USA), and a 24 Fr DrySeal sheath was introduced. Unfractionated heparin was administered at a dose of 100 U/kg, targeting an activated clotting time (ACT) of 300 s.

A Terumo guidewire was advanced from the LBA into the descending thoracic aorta, snared with a gooseneck device, and exteriorized through the DrySeal sheath in the RCFA to establish through-and-through access. A multipurpose A1 (MPA1) catheter was then advanced over this wire from the LBA and exteriorized via the RCFA. The free end of the traction wire attached to the branch of the Castor single-branched stent graft (CSBSG) was introduced into the MPA1 catheter through the RCFA and retrieved from the LBA.

A Lunderquist extra-stiff guidewire (Cook Medical, Bloomington, IN, USA) was inserted into the ascending aorta via femoral access, followed by advancement of the delivery sheath. The CSBSG was rotated to align its branch with the outer curvature of the aortic arch. An angiographic assessment confirmed correct alignment and ensured that the branch traction wire was not entangled.

The stent graft was then carefully advanced and deployed within the arch, with the branch directed into the LSA. During deployment of the main body, systolic blood pressure was pharmacologically reduced to approximately 90 mmHg to enhance placement accuracy. After confirming position under fluoroscopy, the trigger wire was pulled to deploy the main body, followed by deployment of the branch using the traction wire. Once deployment was complete, blood pressure was normalized and angiography confirmed aneurysm exclusion and branch patency.

To prevent a type II endoleak, a vascular plug was delivered via the RBA and deployed at the origin of the ARSA. The RCFA was closed using the previously placed Perclose™ ProStyle™ sutures. Hemostasis at the brachial accesses was achieved with 15 min of manual compression followed by bandaging. The LCFA was closed using the Perclose™ ProStyle™ system.

**Figure 2:**
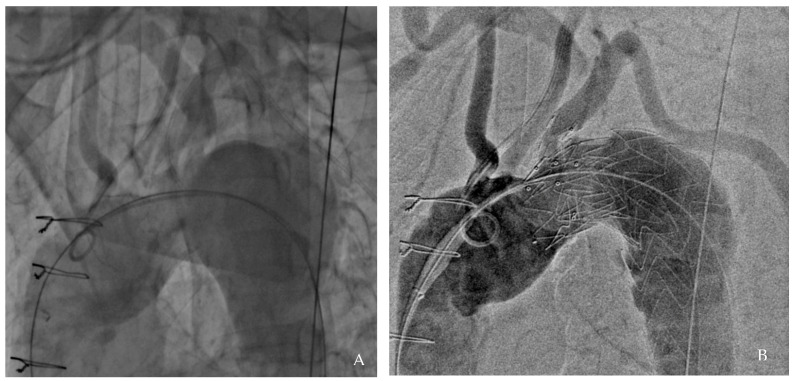
Angiographic results: pre-procedure (**A**) and post-procedure (**B**).

A final angiographic evaluation confirmed successful exclusion of the aneurysm and satisfactory perfusion of the supra-aortic vessels (Figure 2). The patient was discharged five days later on dual antiplatelet therapy (aspirin 100 mg and clopidogrel 75 mg) for three months, followed by lifelong aspirin monotherapy.

**Figure 3:**
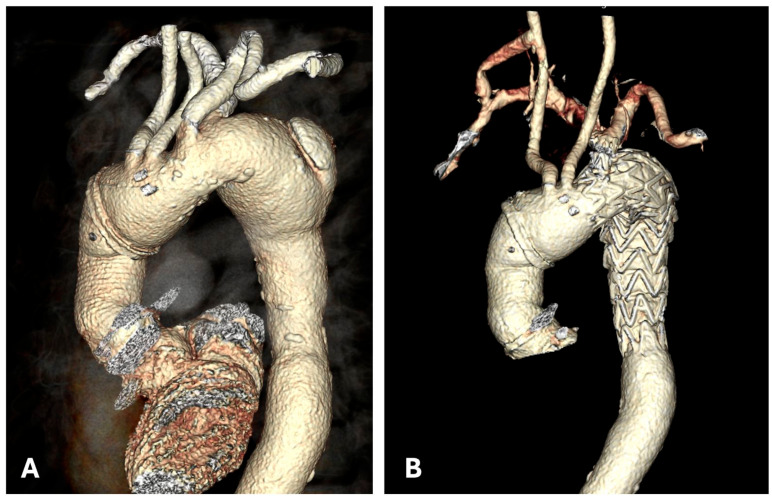
Volumetric rendering visualization via CTA: pre-procedure (**A**) and post-implantation of the prosthesis, carotid-subclavian bypass, and plug placement at the origin of ARSA (**B**).

At the three-week follow-up, the patient was asymptomatic with stable hemodynamics and no complications. Follow-up CTA confirmed optimal procedural results (Figure 3).

## 3. Discussion

This case report highlights a TEVAR performed for a distal arch aneurysm in a patient with an ARSA that originates distal to LSA. Notable aspects of this case include: (1) the use of the CSBSG for aneurysm exclusion, which effectively maintains patency of the LSA; (2) the creation of a right carotid-subclavian bypass involving the ARSA to ensure perfusion to the right upper vascular structures; (3) the implantation of a plug at the origin of the ARSA to prevent type 2 endoleaks. Significant advancements in endovascular stent graft design have facilitated their application in the distal aortic arch and proximal descending aorta [3]. However, the length of the proximal landing zone remains a critical limitation for TEVAR, especially concerning the LSA [4]. Several studies have indicated that covering the LSA can heighten risks of arm ischemia, vertebrobasilar insufficiency, anterior circulation strokes, and paraplegia [5]. Therefore, the European Society for Vascular Surgery guidelines advocate routine LSA revascularization during TEVAR, particularly for patients at high risk of neurological complications [6]. Various techniques for LSA revascularization have been proposed, such as carotid-subclavian bypass, fenestrated devices, chimney techniques, and hybrid approaches [7]. Among them, the Castor single-branched stent graft (Microport Medical) has proven to be highly feasible and efficient, securing an adequate proximal landing zone while preserving necessary LSA perfusion, with favorable outcomes observed at one-year follow-up [8,9].

In our case, the Castor stent graft was employed with the side branch introduced into the LSA to ensure patency and provide an adequate landing zone; the main body was positioned to exclude the aneurysm of the distal arch. The presence of a complex anatomical variant, specifically the ARSA, necessitated the creation of a single right carotid-subclavian bypass to assure perfusion to the right-sided territories. In this instance, the ARSA originated distal to the LSA; therefore, the deployment of the Castor stent graft adequately covered its origin from the arch. To prevent ischemia in the territories supplied by the ARSA, we proceeded with the preventive creation of the aforementioned right carotid-subclavian bypass.

Our team previously described a case involving a double carotid-subclavian bypass followed by the endovascular exclusion of a Kommerell diverticulum and bilateral subclavian artery occlusion in a right-sided aortic arch with an aberrant LSA, although this was executed without utilizing the Castor prosthesis [8].

On the other hand, in the present case to thwart the occurrence of type 2 endoleak a plug was implanted at the origin of the ARSA via the brachial artery, ensuring a secure closure of this anatomical anomaly.

In our scenario an endovascular approach was preferred over open surgery, as the patient had undergone a surgical replacement of the ascending aorta and aortic valve repair two years prior. Engaging in a redo surgical intervention would have represented a higher risk for the patient.

To the best of our knowledge, this is the first case involving the deployment of a single-branched Castor stent graft in a patient with a distal aortic arch aneurysm and ARSA, revascularized through a single right carotid-subclavian bypass.

## 4. Conclusions

This case highlights the value of a customized hybrid approach in the management of distal aortic arch aneurysms, particularly in the presence of complex anatomical variants such as ARSA. By combining surgical revascularization with the use of a CSBSG, it is possible to achieve effective aneurysm exclusion while preserving perfusion to both subclavian arteries. This strategy significantly reduced the risk of neurological and ischemic complications, in line with current guidelines advocating for routine LSA revascularization during TEVAR. To our knowledge, this represents the first documented case employing this specific hybrid configuration for ARSA-associated aortic arch pathology. Our experience underscores the importance of individualized procedural planning and the potential of branched endografts in treating patients with complex aortic anatomy safely and effectively.

## Data Availability

The data presented in this study are available on request from the corresponding author.

## Abbreviations

The following abbreviations are used in this manuscript:

TEVAR	Thoracic Endovascular Aortic Repair
ARSA	Aberrant Right Subclavian Artery
RSA	Right Subclavian Artery
LSA	Left Subclavian Artery
CSBSG	Castor Single-Branched Stent Graft
CTA	Computed Tomography Angiography
RCCA	Right Common Carotid Artery
RCFA	Right Common Femoral Artery
LCFA	Left Common Femoral Artery
RBA	Right Brachial Artery
LBA	Left Brachial Artery
MPA1	Multipurpose A1 (catheter)
ACT	Activated Clotting Time
